# Exogenous Modulation of Retinoic Acid Signaling Affects Adult RGC Survival in the Frog Visual System after Optic Nerve Injury

**DOI:** 10.1371/journal.pone.0162626

**Published:** 2016-09-09

**Authors:** Mildred V. Duprey-Díaz, Jonathan M. Blagburn, Rosa E. Blanco

**Affiliations:** 1 Department of Anatomy and Neurobiology, School of Medicine, University of Puerto Rico, San Juan, PR, United States of America; 2 Institute of Neurobiology, University of Puerto Rico Medical Sciences Campus, San Juan, PR, United States of America; Institut Curie, FRANCE

## Abstract

After lesions to the mammalian optic nerve, the great majority of retinal ganglion cells (RGCs) die before their axons have even had a chance to regenerate. Frog RGCs, on the other hand, suffer only an approximately 50% cell loss, and we have previously investigated the mechanisms by which the application of growth factors can increase their survival rate. Retinoic acid (RA) is a vitamin A-derived lipophilic molecule that plays major roles during development of the nervous system. The RA signaling pathway is also present in parts of the adult nervous system, and components of it are upregulated after injury in peripheral nerves but not in the CNS. Here we investigate whether RA signaling affects long-term RGC survival at 6 weeks after axotomy. Intraocular injection of all-trans retinoic acid (ATRA), the retinoic acid receptor (RAR) type-α agonist AM80, the RARβ agonist CD2314, or the RARγ agonist CD1530, returned axotomized RGC numbers to almost normal levels. On the other hand, inhibition of RA synthesis with disulfiram, or of RAR receptors with the pan-RAR antagonist Ro-41-5253, or the RARβ antagonist LE135E, greatly reduced the survival of the axotomized neurons. Axotomy elicited a strong activation of the MAPK, STAT3 and AKT pathways; this activation was prevented by disulfiram or by RAR antagonists. Finally, addition of exogenous ATRA stimulated the activation of the first two of these pathways. Future experiments will investigate whether these strong survival-promoting effects of RA are mediated via the upregulation of neurotrophins.

## Introduction

Retinoic acid (RA) is a vitamin A-derived lipophilic molecule that plays a major role during early development of the nervous system, setting up dorsoventral and anteroposterior patterning of the neural plate and tube [1]. Its later function is to direct the differentiation of various types of neurons and glia by activating the transcription of many genes, including those that encode transcription factors, cell signaling molecules, enzymes and cell surface receptors [1–5].

Retinoic acid is synthesized by the enzyme retinaldehyde dehydrogenase (RALDH) from retinal [6]. Cellular retinoic acid binding proteins (CRABP-I and CRABP-II) aid in the transport of RA into the nucleus [7], where it binds with its receptors, the retinoic acid receptors (RARs and RXRs). These receptors, of which there are three subtypes, α, β, and γ, belong to the steroid-thyroid hormone receptor family of transcription regulators [8]. RA-bound-RAR and RXR form heterodimers on DNA sequences known as retinoic acid response elements (RAREs) in target gene promoters and activate and enhance transcription of those genes [8]. Recently it was shown that this may also require the presence of the activated FGFR1 receptor [9]. However, evidence has also accumulated for non-canonical, non-genomic effects of RAR, which can apparently activate both the MAPK-CREB and PI3K-Akt pathways directly [10,11], and can also regulate protein translation in postsynaptic dendrites by binding to mRNA [12].

More recently, it has been found that RA signaling also has a role in the CNS of adult animals, particularly in areas of high neuronal plasticity such as the hippocampus, cortex, and striatum, where cells continue to be generated [1,13–17]. It has also been shown to be involved in the control of rhythms within the brain [18]. In addition, defects in RA signaling may occur in various neurodegenerative diseases including ALS [19,20] and Alzheimer’s disease [21,22].

Injury to the adult CNS of mammals is particularly devastating, one reason being that neurons degenerate and die after their axons are damaged, because they are unable to reacquire the trophic support of neurotrophins produced by their synaptic targets [23–26]. The visual system is particularly appropriate for studying how to alleviate these problems because retinal ganglion cells (RGCs) behave like other CNS neurons after injury but are more experimentally accessible. In addition, injury to RGCs is of great medical importance in its own right, causing blindness after optic nerve injury, and in conditions such as glaucoma, diabetes and optic ischemia [27–30].

There is a plethora of studies of the developing CNS that show that RA has stimulatory effects on neurotrophin gene expression and neuronal survival [31–34]. However, there are fewer that investigate whether it also promotes the survival of adult neurons after injury. In cultured slices, RA signaling upregulates BDNF in midbrain dopaminergic neurons via the ERK/MAPK pathway [35,36]. In another, *in vivo*, study the BDNF receptor, TrkB, was upregulated in injured adult rat spinal cord by the RA agonist AM80 [37]. There are a very few studies on survival of RGCs, however, of particular relevance to the present study, RA application reduces apoptosis of RGCs in response to NMDA-induced injury, also via the MAPK pathway [38,39].

The frog visual system is similar to the mammal’s in that many of its RGCs die after axotomy [40], but unlike it in that the surviving RGCs are able to regenerate and reconnect with their targets in the optic tectum [41,42]. Both the survival rate, and regeneration speed, of the frog’s RGCs can be augmented by application of growth factors to optic nerve or eyeball, and we have studied the molecular pathways involved [40,43–46]. All the components of RA signaling are present in the frog retina and tectum, and these are upregulated after optic nerve injury [47].The purpose of the present study is to determine whether application of exogenous RA can augment long-term survival of the RGCs, whether intrinsic RA production also has an influence on survival, and to begin to elucidate the signaling pathways that are involved in these processes.

## Materials and Methods

### Animals

Adult frogs (*Rana pipiens*) of both sexes were used. They were obtained from Connecticut Valley Biological Supply Company (Southampton, MA) and kept in tanks with recirculating tap water at 19°C. This study was carried out in strict accordance with the recommendations in the Guide for the Care and Use of Laboratory Animals of the National Institutes of Health. The protocol was approved by the Institutional Animal Care and Use Committee of the University of Puerto Rico Medical Sciences Campus. All surgery was performed under tricaine anesthesia, and all efforts were made to minimize suffering.

### Surgical technique for optic nerve axotomy

Under 0.3% tricaine anesthesia the right eyeball of series of frogs (*Rana pipiens*) was approached from the palate and an incision made; the extraocular muscles were teased aside and the intraorbital section of the optic nerve was exposed. While avoiding large blood vessels, a hole was made in the meningeal sheath and the nerve within was severed, allowing a narrow, but complete, separation of the stumps within the intact sheath. The incision was sutured and the animal was allowed to recuperate.

### Application of RA signaling agonists and antagonists

The experimental design for this aim is similar to those carried out previously in our lab [44]. RA signaling modulators, either agonist or antagonist, are applied intraocularly as a 10 μL injection 15 mins before optic nerve transection. Stock solutions were first prepared in 10% DMSO and then diluted to a final concentration using 0.1M PBS, allowing for an approximately 1:10 dilution when injected intraocularly. A total of 9 experimental groups, each with 4 animals, were prepared, as follows: (1) control uncut animals, (2) axotomy plus vehicle (PBS-DMSO), (3) axotomy plus the RAR agonist all-trans retinoic acid (ATRA, Sigma, 0.5 μM), (4) axotomy plus RARα agonist (AM80 (Tamibarotene), Tocris, 0.5 μM), (5) axotomy plus RARβ agonist (CD2314, Tocris, 140 nM), (6) axotomy plus RARγ agonist (CD1530, Tocris, 150nM). (7) axotomy plus the RALDH inhibitor disulfiram (Sigma, 50 μM), (8) axotomy plus pan RAR antagonist (Ro-41-5253, Biomol, 4 μM), and (9) axotomy plus RARβ antagonist (LE135, Tocris, 2 μM). Animals were allowed to recover, and received a second intraocular application after one week. In initial pilot experiments, the agonists and antagonists were tried at concentrations recommended by the manufacturer, and as used in other published studies. If an effect on survival was observed, we also tried lower and higher concentrations to find the optimal doses. In general, the effective dosages used in this study are lower than the manufacturer’s recommended concentration.

### TDA dextran amine retrograde labeling of RGCs

A 1 mm square piece of cellulose acetate membrane filter (0.8 μm pore size; Schleicher and Schuell, Keene, NH) was infused with a saturated solution of Texas Red dextran amine (TDA; 3,000 MW; Molecular Probes) in ethanol and left to air-dry so that particles of TDA were embedded within the filter. The optic nerve of control or experimental animals was exposed and cut close to the back of the eyeball. The piece of filter was inserted into the stump and the palate was sutured. After 48 − 60 hours, the frog was anesthetized, and fixed. With these parameters, TDA consistently labeled 80 − 84% of the cells in the GCL of control retinas. The percentage of displaced amacrine cells in *Rana pipiens* retina has been previously calculated as 16% [48], so we appear to be labeling most of the RGCs. The percentage of total cells in the GCL labeled with TDA decreased with time after axotomy; this was not due to a reduced efficacy of the retrograde labeling procedure but corresponded to the expected decline in RGC numbers at these times.

### Quantification of cells in the ganglion cell layer

Four untreated animals and four animals per each experimental condition were used for the RGC long-term survival assay. Animals were anesthetized and their hearts exposed for perfusion with frog Ringer solution and later with 10–15 mL of ice-cold 4% paraformaldehyde in 0.1M PBS (pH 7.3). The retinas were dissected out and post-fixed for 2 to 3 h in fixative buffer at room temperature, then washed in phosphate buffer. The retinas were then flattened, axon layer uppermost, onto a gelatinized microscope slide and mounted in Vectashield (Vector Laboratories, Burlington, CA, USA). All retinas used for counting had total areas of 70–80 mm^2^. Cell counts in the GCL were made from two regions, located in the inferior quadrant (1 mm from the optic nerve) and the temporal quadrant (two-thirds the distance to the periphery). Images were captured, from the areas mentioned above, with a x40 objective on a Zeiss Axioskop LSM5 Pascal laser scanning confocal microscope. Each sample area was 0.05mm^2^.

### Total protein isolation from retinal tissue

At least three extractions from control and experimental tissues were obtained from two animals each per pool. Isolated tissue was homogenized in lysis buffer containing 10 mM Tris-HCl pH 7.6, 150 mM NaCl, 0.5% Nonidet P-40, 1 mM EDTA, 0.2 mM phenylmethylsulfonyl fluoride, 1/100 per volume of protease inhibitor cocktail (0.1 μg/mL leupeptin, 0.001 μg/mL pepstatin, 0.1 μg/mL aprotinin), and 1/100 per volume of phosphatase inhibitor cocktail I and II (Sigma) using a motorized homogenizer. Cells were disrupted by sonication for 10 s (1 pulse per s at maximum power) using a Sonic Dismembrator (Fisher Scientific) at 4°C. Samples were then left to stand for 30 min at 4°C and centrifuged at 14,000 rpm for 15 min at 4°C. Protein concentration was determined using a Lowry-based assay from Bio-Rad (DC-protein assay; Bio-Rad) and read in a spectrophotometer.

### Western blotting

Proteins were separated by sodium dodecyl sulfate-polyacrylamide gel electrophoresis. Approximately 50 μg of total protein from each sample was separated in a 4–20% gel (Bio-Rad). Electrophoresed proteins were then transferred to a polyvinylidene difluoride membrane (Millipore) for 30 min and blocked for 1 h. After blocking, the membranes were incubated overnight at 4°C with the following rabbit antibodies (all obtained from Cell Signaling Technology and used at 1:1000 dilution): (1) phosphorylated p44/p42 MAPK (cat# 4370), (2) total p44/p42 MAPK (cat# 9102), (3) phosphorylated AKT (cat# 4060) and (4) total AKT (cat# 4691), (5) phosphorylated STAT3 (cat# 9145) and (6) a mouse anti total STAT3 (cat# 9139), (7) a rabbit anti-GAPDH (1:3000, Novus Biologicals, cat# NB300-327) diluted in blocking solution. Several of these antibodies have been shown by the manufacturers to be specific for their cognate proteins in a range of vertebrate phyla including zebrafish (1, 2, 3), and in *Drosophila* (1, 3, 4, 7) and yeast (1, 2, 7). For STAT3, BLAST analysis of the antigenic region shows 100% identity with *Xenopus* and zebrafish. In addition, specificity of staining was tested by omitting the primary antibody and by preabsorption with their corresponding peptides, which resulted in the absence of all immunostaining. Bound primary antibody was detected using a peroxidase-conjugated goat anti-rabbit or mouse secondary antibody (1:2000, Bio-Rad) for 2 h at room temperature. To visualize immunoreactive bands, membranes were developed for analysis using a chemiluminescence method (WesternSure ECL substrate kit, Li-Cor Biotech). Each set of Western blots was performed in duplicate for each one of the protein extractions. Images from the processed membranes were captured using the ISO400R Kodak Image Station Software (Kodak) and analyzed using the Image J program (Wayne Rasband, NIH). Relative intensities for protein signals were averaged from duplicates and normalized against the average non-phospho-protein or GAPDH values, then standardized to average control values.

### Statistical Analysis

The mean values of experimental measurements were plotted in bar charts, with error bars representing the S.E.M. One-way ANOVA and *post-hoc* Tukey tests (or Tukey-Kramer in the case of unequal sample numbers) were performed to determine statistically significant changes.

### Immunohistochemistry

The eye cups of four animals (N = 4) were fixed with buffered 2% paraformaldehyde solution for 1 h, washed with phosphate-buffered saline, and then placed in 30% sucrose for cryoprotection at 4°C overnight. After being frozen, cryostat sections of 12 μm from both control (uncut and untreated) and experimental eyes were cut and placed on the same slide, yielding approximately 15 slides. For immunocytochemistry, the sections were washed once for 30 min in phosphate-buffered saline (PBS) solution, and later incubated for 1 h in 10% normal goat serum. Tissue was then incubated overnight with rabbit polyclonal antisera (all from Cell Signaling Technology) against phosphorylated p42/44 MAPK (ERK1/2), phosphorylated AKT, and phosphorylated STAT3 antibodies, all at a dilution of 1:200 in 0.1 M PBS + 0.3% Triton X-100 + 0.5% bovine serum albumin. After several washes with 0.1 M PBS the sections were incubated with goat anti-rabbit or anti-mouse IgG-CY3 (1:100, Jackson Immunoresearch Labs) for 2 h at room temperature. The sections were rinsed in PBS six times for 10 min each, and mounted in Polymount. Specificity of staining was tested by omitting the primary antibody and by preabsorption with their corresponding peptides, which resulted in the absence of all immunostaining. In order to try to ensure consistent antibody staining, frozen sections from control animals and from animals at different experimental times were processed together, using the same antibody dilutions. Preparations were viewed and images acquired using a Zeiss Pascal, LSM 510 Meta Confocal Laser microscope (Carl Zeiss Inc), using the same objective lens (x40) and confocal settings for the different treatments. Detector gain was set so that the brightest pixels in the image of the most intensely-stained section on a slide were not saturated, then maintained constant throughout. Control and experimental sections were imaged with the same settings. For the immunohistochemistry figure, images were all given the same adjustment for maximal contrast, by setting the black point to the darkest pixel in the space below the retina, and ensuring that the white point was the brightest pixel in the image, either of the immunostaining where it was most intense or, when immunostaining was faint, of the brightest of the scattered punctate debris clusters. With these minor adjustments, background tissue staining (the lowest staining regions of the IPL or the ONL) was consistently about 10% of the maximum brightness. Plates were assembled using Adobe Photoshop (Adobe Systems, Inc.) and CorelDraw (Corel Corp.).

## Results

### Retinoic acid signaling promotes the long-term survival of axotomized RGCs

**Fig 1:**
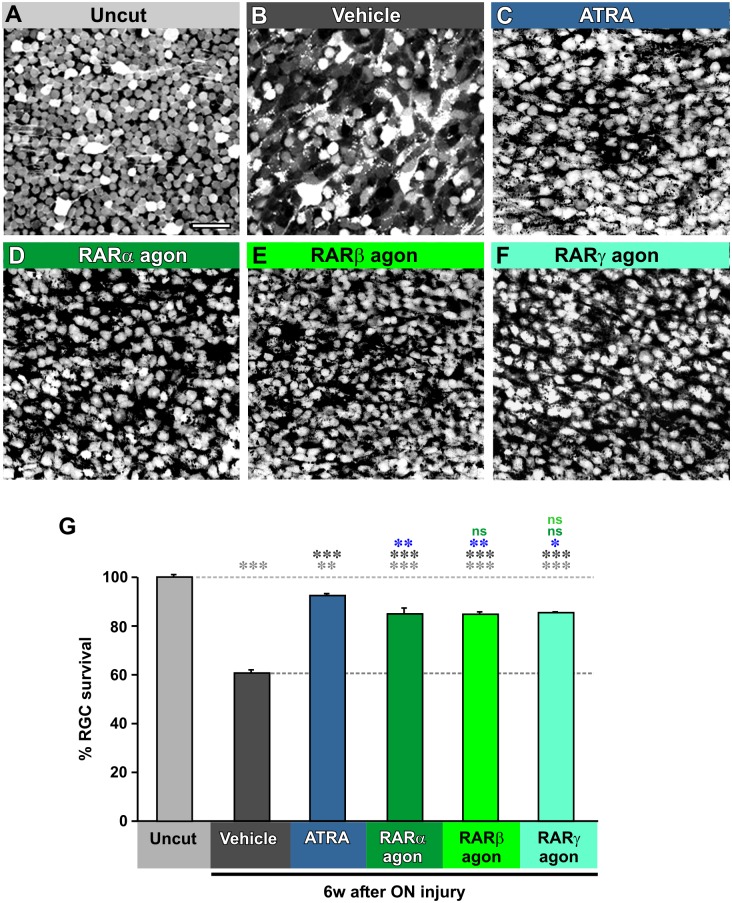
Retinoic signaling agonists promote long-term survival of axotomized RGCs. **A-F.** Representative fields of whole-mount retinas in which RGCs have been retrogradely labled with Texas Red dextran amine (TDA). **A.** Uncut normal retina. **B.** Retina from animal at 6 weeks after axotomy, eye injected with PBS and DMSO (vehicle). **C.** Retina from animal at 6 weeks after axotomy, eye injected with PBS and DMSO with the addition of all-trans retinoic acid (ATRA). **D.** Retina from animal at 6 weeks after axotomy, eye injected with PBS and DMSO with the addition of the RARα agonist AM80 (RARα agon). **E.** Retina from animal at 6 weeks after axotomy, eye injected with PBS and DMSO with the addition of the RARβ agonist CD2314 (RARβ agon). **F.** Retina from animal at 6 weeks after axotomy, eye injected with PBS and DMSO with the addition of the RARγ agonist CD1530 (RARγ agon). **G.** Quantification of RGC percentage survival in the temporal region of the retina at 6 weeks after axotomy. Vehicle-treated retinas show a significant 40% decrease in RGC numbers. Treatment with ATRA or RAR agonists rescues many of these RGCs. One-way ANOVA followed by *post-hoc* Tukey tests: * p < 0.05, ** p < 0.01, *** p < 0.001, N = 4 animals in all cases. Error bars represent the S.E.M. Scale bar in A = 50 μm.

The first objective of this study was to assay whether, and to what extent, RA application affected RGC survival after nerve injury. To this end we used an assay that has been published previously [40,44] to count the number of surviving RGCs that had been labeled retrogradely with TDA at 6 weeks after axotomy (Fig 1A–1F). This method reliably labels 80–84% of the cells in the ganglion cell layer of the retina [44], and, since displaced amacrines may make up the remaining 16% [48], we are fairly confident that all the RGCs are labeled. Axotomy-induced RGC death in frogs occurs at later times compared to higher vertebrates, usually a month after the injury [49,50].

Cutting the nerve, along with treatment with PBS and DMSO (vehicle) reliably resulted in an approximately 40% decrease in RGC numbers by 6 weeks (p = 0.00012, Fig 1G), as also found in the previous two studies [40,44]. An injection of all-trans retinoic acid (ATRA) into the eye at the time of axotomy, followed by a second injection a week later, resulted in the survival of many more cells, with an approximately 53% increase compared to treatment with PBS and DMSO (vehicle) alone (p = 0.00012) This represents a very substantial (92%) long-term recovery of RGC numbers as compared to unoperated animals (p = 0.0077).

We were interested to find out which RAR types might be involved in mediating this survival response so we applied the agonist AM80, which, at the concentration we used (0.5 μM), is specific for RAR type α [51]. Like ATRA, this also strongly promoted RGC survival, although it was somewhat less effective (85% survival versus 92%, p = 0.00012). Similarly, the RARβ agonist CD2314 and the RARγ agonist CD1530 also each promoted the survival of approximately 85% of the RGCs (p = 0.00012 for both) (Fig 1G). These results suggest that no single RAR type is required for RA-promoted RGC survival, rather that all three may play a role.

**Fig 2:**
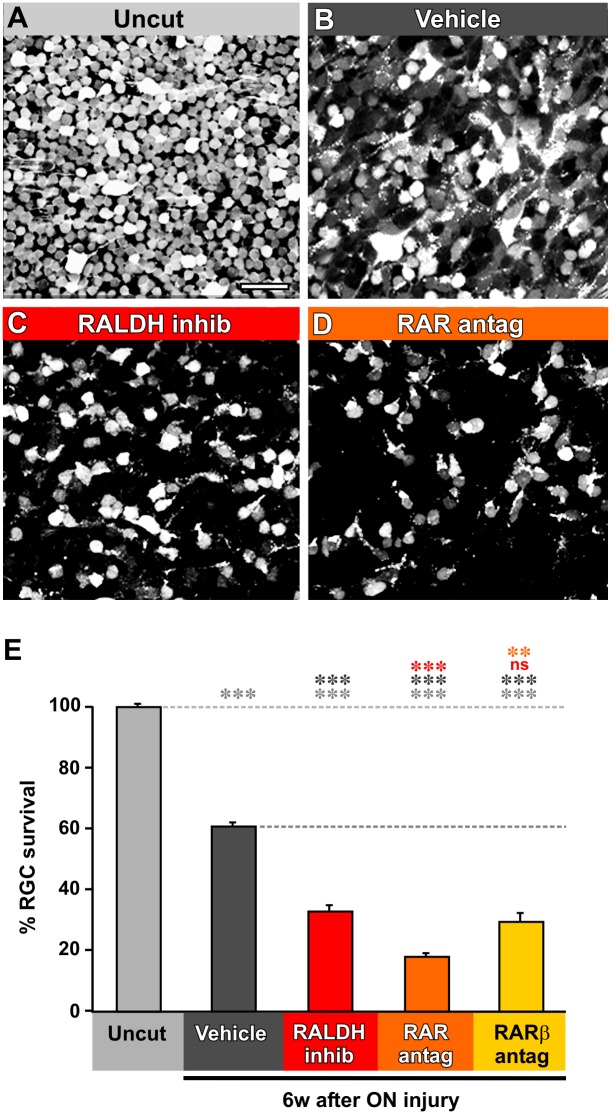
Retinoic signaling antagonists reduce long-term survival of axotomized RGCs. **A-D.** Representative fields of whole-mount retinas in which RGCs have been retrogradely labled with Texas Red dextran amine (TDA). **A.** Uncut normal retina. **B.** Retina from animal at 6 weeks after axotomy, eye injected with PBS and DMSO (vehicle). **C.** Retina from animal at 6 weeks after axotomy, eye injected with PBS and DMSO with the addition of the RALDH inhibitor disulfiram (RALDH inhib). **D.** Retina from animal at 6 weeks after axotomy, eye injected with PBS and DMSO with the addition of the pan-RAR antagonist Ro-41-5253 (RAR antag). **E.** Quantification of RGC percentage survival in the temporal region of the retina at 6 weeks after axotomy. Vehicle-treated retinas show a significant 40% decrease in RGC numbers. Treatment with RALDH inhibitor further reduces survival. Only the antagonist against RAR, not RARβ, further reduces survival. One-way ANOVA followed by *post-hoc* Tukey tests: * p < 0.05, ** p < 0.01, *** p < 0.001, N = 4 animals in all cases. Error bars represent the S.E.M. Scale bar in A = 50 μm.

We hypothesized that native RA synthesis could also affect the number of RGCs that are able to survive axotomy, in which case blocking the synthetic enzyme would further reduce their numbers. Indeed, application of the RALDH inhibitor disulfiram reduced the number of RGCs to 53% of those which would normally have survived (p = 0.00012) (Fig 2E). We tested whether this subpopulation of RGCs that require endogenous RA for survival required RARs to mediate this effect by applying the pan-RAR antagonist Ro-41-5253, or the specific RARβ antagonist LE135. The pan-RAR antagonist reduced survival even further (Fig 2E), down to 30% of what it would have been with axotomy and vehicle treatment alone (p = 0.00012), and to 54% of the value with RALDH inhibition (p = 0.00043). However, the RARβ antagonist did not significantly reduce survival more than did RALDH inhibition (p = 0.708). These results suggest that the subpopulation of RGCs that depend on endogenous RA for survival do require RARs, some which are of the β subtype.

### Axotomy-induced MAPK signaling is activated by, and dependent upon, retinoic acid

**Fig 3:**
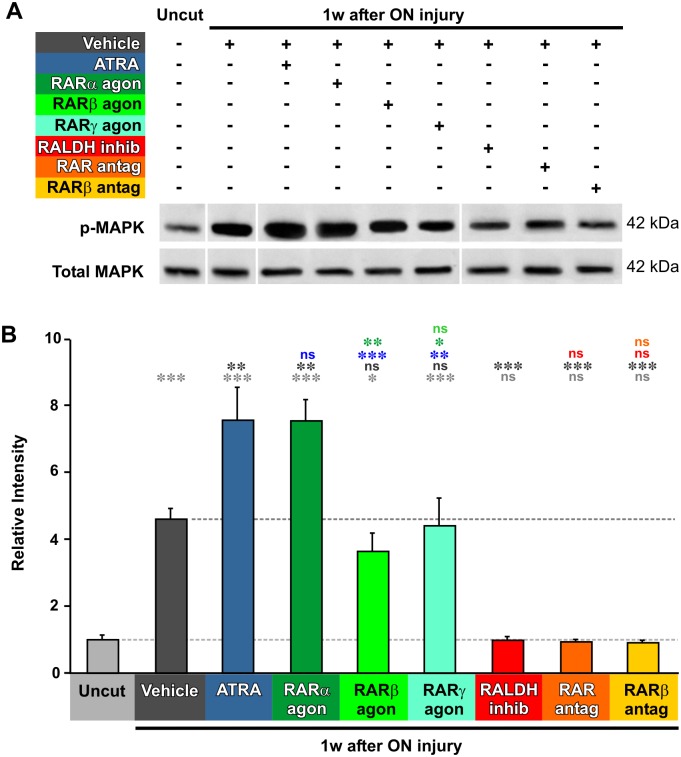
Axotomy-induced MAPK activation is dependent on RA. **A**. Representative Western blots of phosphorylated-MAPK and total MAPK from extracts of retinas from uncut animals, and animals at 1 week after axotomy. Approximate molecular weight in kDa is indicated. The order of some of the bands was rearranged to correspond to the bar chart below. **B.** Quantification of Western blots, standardized to control values. pMAPK levels are increased at 1 week after axotomy, with a further increase elicited by ATRA or RARα agonist application. Inhibition of RA synthesis, or application of RAR antagonists, prevents the axotomy-induced increase in pMAPK. One-way ANOVA followed by *post-hoc* Tukey-Kramer tests: * p<0.05, ** p<0.01, *** p<0.001, N = 4, 4, 3, 3, 3, 3, 3, 4, 4, and 3 pools, each of 2 animals and with duplicate measurements. Error bars represent the S.E.M.

What are the signaling pathways that are initially activated by RA application, and which might therefore be involved in mediating its survival effect in the long term? In previous studies we have found that axotomy provokes an early increase in MAPK signaling [43], with a doubling of the phosphorylated (activated) enzyme at 1 week after axotomy. Here we repeated these Western blotting experiments, using more sensitive techniques, and found an even larger increase in activated MAPK after axotomy (an almost 4.5-fold increase: Fig 3, p = 0.0002). It should be noted that, in frogs, only the single ERK2 isoform (42 kDa) is present [43,52]. Injection of ATRA at the time of axotomy further increased MAPK activation by 64% (p = 0.0043), as did the RARα agonist AM80 (p = 0.0046). On the other hand, the RARβ and RARγ agonists had no significant effect (p = 0.8562, p = 0.9999). Prevention of endogenous RA synthesis with the RALDH inhibitor completely eliminated the axotomy-induced activation of MAPK signaling (Fig 3B) (p = 0.0002), as did blocking the RA response with RAR and RARβ antagonists (p = 0.0002, p = 0.0004). These results indicate that endogenous RA signaling, mediated via RARβ receptors, is necessary for the axotomy-induced activation of MAPK in the retina. The addition of more RA to the eye further stimulates MAPK activation, and this effect is mediated via RARα receptors.

### Axotomy-induced AKT signaling is dependent upon retinoic acid but is not stimulated by it

**Fig 4:**
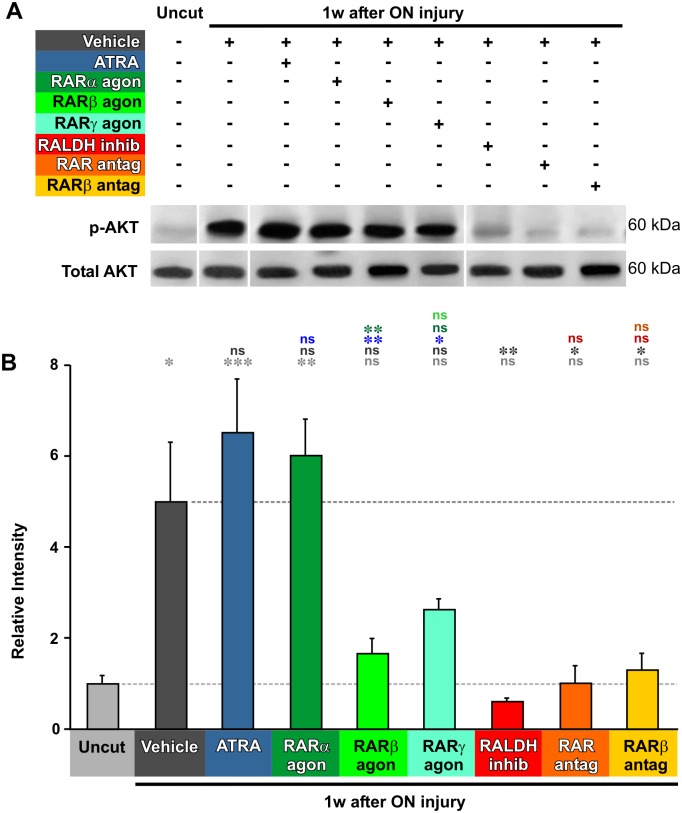
Axotomy-induced AKT activation is dependent on RA. **A**. Representative Western blots of phosphorylated-AKT and total AKT from extracts of retinas from uncut animals, and animals at 1 week after axotomy. Approximate molecular weight in kDa is indicated. The order of some of the bands was rearranged to correspond to the bar chart below. **B.** Quantification of Western blots, standardized to control values. pAKT levels are increased at 1 week after axotomy. Application of ATRA or other RAR agonists do not significantly increase the levels of pAKT further. Inhibition of RA synthesis, or application of RAR antagonists, prevents the axotomy-induced increase in pAKT. One-way ANOVA followed by *post-hoc* Tukey tests: * p<0.05, ** p<0.01, *** p<0.001, N = 3 pools in all cases, each of 2 animals and with duplicate measurements. Error bars represent the S.E.M.

Quantification of the AKT signaling pathway by measurement of phospho-AKT levels showed a very strong, approximately 5-fold increase at 1 week after axotomy (Fig 4) (p = 0.0154). In contrast to MAPK however, the axotomy-induced AKT signaling was not significantly potentiated by ATRA or by any of the RAR agonists (p = 0.8194, p = 0.0594, p = 0.3277). Similarly to MAPK, the axotomy-induced increase in AKT signaling was dependent on intrinsic RA, being inhibited by RALDH inhibition (p = 0.0067) and both the pan-RAR and RARβ antagonists (Fig 4B) (p = 0.0158, p = 0.0290).

### Axotomy-induced STAT3 signaling is activated by, and dependent upon, retinoic acid

**Fig 5:**
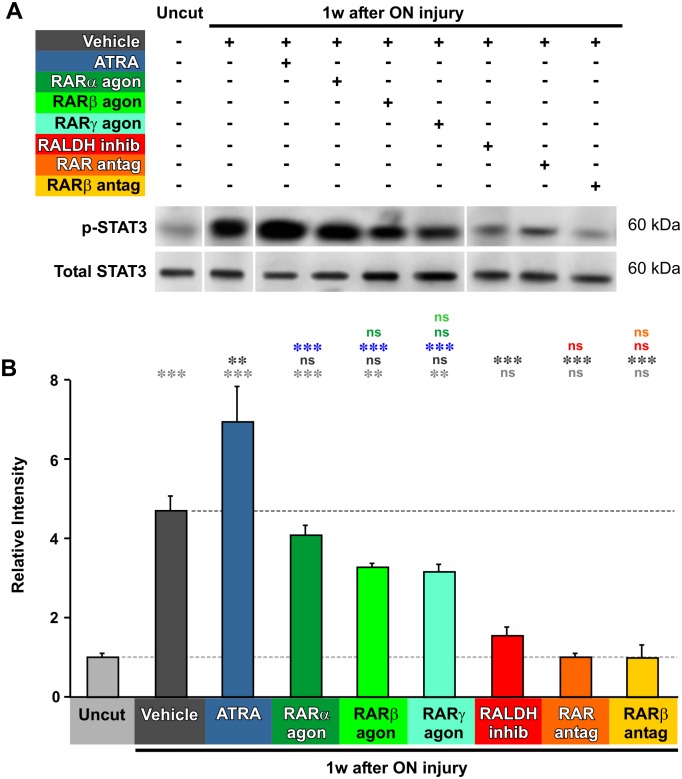
Axotomy-induced STAT3 activation is dependent on RA. **A**. Representative Western blots of phosphorylated-STAT3 and total STAT3 from extracts of retinas from uncut animals, and animals at 1 week after axotomy. Approximate molecular weight in kDa is indicated. The order of some of the bands was rearranged to correspond to the bar chart below. **B.** Quantification of Western blots, standardized to control values. pSTAT3 levels are increased at 1 week after axotomy. Application of ATRA increases the levels of pSTAT3 further, but other RAR agonists do not. Inhibition of RA synthesis, or application of RAR antagonists, prevents the axotomy-induced increase in pSTAT3. One-way ANOVA followed by *post-hoc* Tukey-Kramer tests: * p<0.05, ** p<0.01, *** p<0.001, N = 4, 4, 3, 3, 3, 3, 3, 4, 4, and 3 pools, each of 2 animals and with duplicate measurements. Error bars represent the S.E.M.

Similarly to the other pathways, quantification of STAT3 signaling showed a 4.7-fold increase at 1 week after axotomy (Fig 5) (p = 0.0001). Similar to MAPK, this increased STAT3 signal was potentiated 50% by injection of ATRA (p = 0.0064), but none of the RAR agonists showed this effect (p = 0.9468, p = 0.1743, p = 0.1162). As above, the axotomy-induced increase in STAT3 signaling was almost abolished by RALDH inhibition (p = 0.0002), and by application of RAR antagonists (Fig 5) (p = 0.0001, p = 0.0001).

### Increased activity of signaling pathways is localized to RGCs

**Fig 6:**
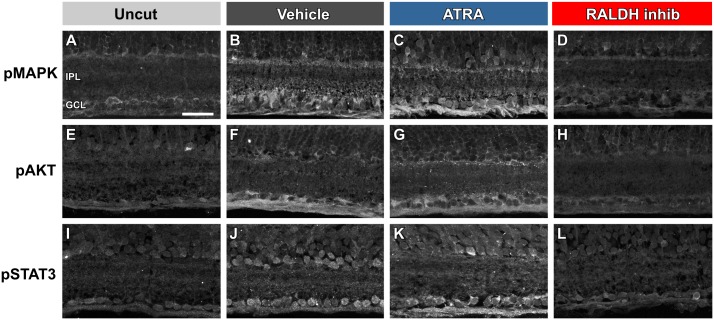
Increased activity of signaling pathways is localized to RGCs. Immunostaining was carried out against pMAPK, pAKT and pSTAT3 in frozen sections of retinas from uncut animals (A, E, I), animals at 1 week after the optic nerve was cut and the eyeball injected with vehicle (B, F, J), animals at 1 week after the optic nerve was cut and the eyeball injected with ATRA (C, G, K), and animals at 1 week after the optic nerve was cut and the eyeball injected with RALDH inhibitor (D, H, L). In A, the inner plexiform layer (IPL) and ganglion cell layer (GCL) are indicated. **A, E, I.** In control frog retinal sections, low levels of pMAPK, pAKT and pSTAT3 immunoreactivity are present in the GCL and the INL. **B, F, J.** One week after axotomy, immunostaining of all three proteins has increased in intensity, particularly in the cell bodies of RGCs of the GCL and their axons, and in the cells in the INL. **C, G, K.** One week after axotomy and ATRA treatment, immunostaining of all three proteins is intense in the cell bodies of RGCs of the GCL and their axons, and in the cells in the INL. **D, H, L.** One week after axotomy and RALDH inhibitor treatment, faint immunostaining of all three proteins is present in the GCL and the INL. Scale bar in A: 50 μm.

The above Western blot results show that MAPK, AKT and STAT3 signaling pathway activity is increased in the retina after axotomy and modulated by various treatments. In order to confirm that these increases took place in the RGCs themselves, we examined immunostaining in retinal frozen sections using the phospho-protein antibodies (Fig 6). Low levels of pMAPK, pAKT and pSTAT3 are normally present in RGCs in the ganglion cell layer (GCL) and in some inner cells of the inner nuclear layer (INL) (Fig 6A, 6E and 6I). One week after axotomy, this immunostaining is more intense (Fig 6B, 6F and 6J), and is also strong after ATRA injection (Fig 6C, 6G and 6K). Finally, injection of the RALDH inhibitor disulfiram reduces immunostaining of these proteins to low levels (Fig 6D, 6H and 6L).

## Discussion

Previously, we have demonstrated that the neurotrophic factors FGF-2 and BDNF are normally present at low levels in the frog retina and tectum, and that they are upregulated in response to axotomy [53,54]. Exogenous application of these factors to the eyeball or nerve promotes the long-term survival of axotomized RGCs, and we have made progress in characterizing the intracellular signaling pathways that are involved [40,43–45].

Retinoic acid (RA) is an important morphogen that controls the patterning and differentiation of neurons during development, and it has also been identified as a signaling molecule and transcriptional activator that regulates the expression of target genes, including those of growth factors and their receptors [35,36,55–58]. We now have similar evidence that RA and its synthetic and degradative enzymes are likewise present at low levels in the frog visual system and are upregulated after nerve injury [47]. In the present study we investigate the effects on long-term RGC survival of manipulating RA levels in the eyeball, either by applying it (or synthetic analogues thereof) exogenously, or by inhibiting endogenous RA production.

### RA is a particularly effective promoter of long-term RGC survival

Injection of low doses of the RAR-specific agonist all-trans retinoic acid (ATRA) is very effective at promoting the 6-week survival of more than 90% of the axotomized RGCs. In comparison, two applications of FGF-2 to the cut nerve achieve a survival rate of barely 80%, and intraocular application is ineffective [44]. The synthetic agonists AM80, CD2314 and CD1530, which are more specific for the RAR receptor types, namely RARα, RARβ and RARγ respectively, each promoted the survival of approximately 85%. This suggests no single RAR type is responsible for mediating RA-promoted RGC survival, rather that all three may play a role.

RA has been used to increase neuronal survival in a few other systems, in particular the dopaminergic midbrain neurons that die in Parkinson’s disease [36,59], where it also appears to rescue about 90% of the cells that would have died. RARβ receptor activity is associated with protection of motorneurons from cell death associated with oxidative injury [20]. RA increases the survival of perinatal rat cholinergic (but not GABAergic) spinal cord neurons [60]. RA, acting through RARs, significantly protects hippocampal neurons from oxygen/glucose-deprivation-mediated cell death [61]. Of more direct relevance to our study, intraocular injections of RA in mice with diabetic retinopathy reduced to a quarter the number of apoptotic cells throughout the different layers of the retina, including the GCL [38]. Similarly, ATRA reduced NMDA-induced apoptosis of RGCs, and this effect was prevented by a MAPK kinase inhibitor [39].

Synthetic agonists of RA receptors are more stable and allow the function of different RAR types to be probed more specifically [62]. Our experiments using these agonists indicate that all three RAR types, α, β and γ, have similar effects on RGC survival. In other systems, the agonist AM80 (which preferentially activates RARα) has been associated with neuroprotective effects [36,63–65]. On the other hand, RARβ agonists improve axonal outgrowth [66,67]. While RARγ is involved in early development and neuronal proliferation [68,69], its expression in the CNS is limited [70]. It does however appear to inhibit apoptosis of neuroblastoma cells, via an interaction with RARβ [71].

Despite the fact that the actions of RA in the nucleus are mediated by heterodimers of both RAR and RXR [8], in this study we have focused specifically on the former, using ATRA to activate RARs specifically [72–74]. In mouse, at least, ATRA is the only endogenous ligand necessary for retinoid signaling, and the RXR ligand, 9-cis-RA, is undetectable [75]. In fact, the endogenous ligand for RXRs has remained elusive until recently [76].

### Endogenous retinal production of RA is important for RGC survival after axotomy

We have shown here that reducing endogenous retinal RA activity by inhibiting its synthesis, or by blocking its receptors, dramatically decreases the number of surviving RGCs. Thus, at least in this amphibian model system, RA signaling is critical for the survival of the RGC population that would normally stay alive after nerve injury.

We found that injection of the widely-used RALDH inhibitor, disulfiram, further halves long-term RGC survival after axotomy, suggesting that intrinsic RA synthesis within the retina helps the neurons survive axotomy. A potential complication with interpreting these effects is that the active metabolite of disulfiram can act on other targets [77,78], including acting as an NMDA receptor antagonist [79]. However, this result is mimicked by the RAR antagonists, with the pan-RAR antagonist showing an even stronger effect. Thus, the result that disulfiram reduces RGC survival is most likely to be due to a reduction in RA synthesis, rather than inhibition of NMDA receptors. We also have immunohistochemical evidence that both the synthetic enzyme RALDH and the degradative enzyme CYP26A1 are localized together in RGCs, suggesting that RA synthesis by these neurons has an autocrine effect [47]. Our present results suggest that this autocrine effect is mediated by RARs, in particular RARβ, which are also localized in the RGCs, and are upregulated by axotomy [47]. Activation of RARs in the RGCs would then increase their chances of survival in the face of axotomy, via distinct intracellular signaling pathways.

Numerous studies have shown the relevance of the endogenous RA signaling in other areas of the nervous system. RA deficiency not only significantly impairs learning and spatial memory as a consequence of altered synaptic plasticity [12,80–84], but also lowers the ability of new neurons to survive in the adult hippocampus, and thus compromises the process of neurogenesis which is relevant for memory function [80,85]. Disulfiram inhibition of RA synthesis disrupts the process of song maturation in the brain of adult zebra finches [86,87].

We show here that inhibition of RARs further decreases RGC survival after axotomy, causing cells to die that would otherwise have survived. RAR antagonists have similar effects in other systems. LE135, the selective RARβ antagonist used in the present study, decreases RA’s neuroprotective effects in studies of Alzheimer’s disease [88]. Inhibition of RARs using the pan-RAR antagonist Ro 41–5253 has been shown to downregulate the expression of other RA signaling components, such as CRABPII and RARβ [89], and dominant negative RAR expression in mouse olfactory neurons results in increased cell death [90].

### Differential involvement of MAPK, AKT, and STA3 signaling in mediating the response to RA

In view of the fact that the MAPK pathway has been implicated in the survival-promoting effects of RA application in both RGCs and midbrain neurons [35,39], we proceeded to test the activities of that pathway, and also the PI3-AKT and STAT3 pathways, in frog retina after axotomy and manipulation of RA levels. We found that all three signaling pathways are strongly activated by 1 week after axotomy, to approximately the same extent (a 5-fold increase in phosphorylated protein). We have seen such an increase in MAPK (although not of this magnitude) before in earlier studies [44], although the different antibodies and more sensitive detection methods we use here probably explain the larger changes that were observed.

Application of ATRA further increases MAPK and STAT3 activity (but not that of AKT) and, in the case of MAPK, this is mimicked by the predominantly RARα agonist AM80. The effects of blocking the production of endogenous RA, or the activity of RARs, are even more dramatic—both treatments completely abolish the axotomy-induced upregulation of all three pathways.

In the chick embryonic retina ATRA application elevates MAPK signaling 24 h later, but not AKT or STAT3 [91]. ATRA causes rapid MAPK/ERK activation in neuronal cultures, which leads to CREB phosphorylation [10,92]. This RA-RAR interaction is thought to be non-genomic, due to its rapidity and lack of requirement for RAREs in target genes [10]. In hippocampal neuronal cultures ATRA activates membrane-bound RARα, and then the ERK/MAPK pathway, stimulating protein translation and dendritic growth [92]. We saw no evidence of recruitment of the PI3-AKT pathway by ATRA application, although in neuroblastoma cells RA activates that pathway by a similar rapid non-genomic mechanism [11]. The present study appears to be the first report of STAT3 being activated by ATRA application in neurons.

In our previous study, the survival effect of FGF-2 application appears, at least in major part, to be due to its upregulation of synthesis of both BDNF and its receptor TrkB [44]. At least in the short term (24h – 2 week), BDNF and TrkB upregulation by FGF-2 requires the MAPK/ERK pathway and CREB phosphorylation [45]. AM80 stimulates BDNF synthesis in dopaminergic neurons in cultured slices of rat midbrain, via a complex sequence of events in which it acts via RARs to first increase nitric oxide synthase expression, then cyclic GMP production and PKG activation which, in turn, upregulates MAPK signaling which, via binding of CREB, upregulates *bdnf* mRNA expression [35]. Regarding the other signaling pathways, inhibition of PI3-AKT signaling blocks the protective effect of AM80 [36] but does not prevent the increase in *bdnf* mRNA [35]. In the future, it will be of great interest to determine whether ATRA application also causes upregulation of BDNF synthesis in axotomized frog RGCs.

## Conclusions

We have shown here that long-term survival of axotomized RGCs is returned to almost normal levels by treatment with RA, or with agonists for its receptors. Endogenous production of RA is also critical for the survival of some of the axotomized neurons, along with the activity of RA receptors, particularly those of the RARβ subtype. Axotomy activates the MAPK, STAT3 and AKT pathways; this activation is dependent on endogenous RA, and the addition of RA stimulates the activation of the first two of these pathways. In the future, it will be important to investigate whether these survival-promoting effects of RA are mediated via the upregulation of growth factors such as BDNF, and whether the CREB pathway is involved.
